# Relationship between Axial Length and Levels of TGF-*β* in the Aqueous Humor and Plasma of Myopic Patients

**DOI:** 10.1155/2021/8863637

**Published:** 2021-02-26

**Authors:** Yuwen Hsiao, Yiting Cao, Yu Yue, Jibo Zhou

**Affiliations:** ^1^Department of Ophthalmology, Shanghai Ninth People's Hospital, Shanghai Jiao Tong University, School of Medicine, Shanghai 200011, China; ^2^Shanghai Key Laboratory of Orbital Diseases and Ocular Oncology, Shanghai 200011, China; ^3^Department of Ophthalmology, Shanghai Aier Eye Hospital, Shanghai 200336, China; ^4^Department of Ophthalmology, First Affiliated Hospital of Chongqing Medical University, Chongqing Key Laboratory of Ophthalmology, Chongqing Eye Institute, Chongqing 400016, China; ^5^Chongqing Branch of National Clinical Research Center for Ocular Disease, Chongqing 400016, China

## Abstract

**Purpose:**

To investigate the levels of transforming growth factor-*β* (TGF-*β*) in human aqueous humor (AH) and plasma (PL) of patients with myopia, and verify whether there is an association between these levels and their association with axial length (AL).

**Methods:**

Thirty-eight myopic patients who received intraocular collamer lens (ICL) implantation were enrolled in this cross-sectional study. Patients were divided into three groups based on AL with cut-off points of 26 and 28 mm. AH and PL samples were obtained during ICL implantation surgery. The levels of TGF-*β*1, TGF-*β*2, and TGF-*β*3 in the AH and PL samples were measured using Luminex xMAP Technology kits (Milliplex xMAP kits). The protein levels of TGF-*β*s in both AH and PL samples and their relationships with AL were analyzed.

**Results:**

In all, 38 patients (59 eyes) were enrolled and divided into the three groups: group A contained 7 people (10 eyes), group B contained 22 people (37 eyes), and group C contained 9 people (12 eyes). In the AH group, we detected TGF-*β*1 (*P*_50_: 19.97 pg/mL), TGF-*β*2 (2446.00 pg/mL), and TGF-*β*3 (26.33 pg/mL); in PL, these concentrations were 8984.00, 523.44, and 210.47 pg/mL, respectively. The levels of TGF-*β*1 and TGF-*β*3 in AH were positively associated with AL. None of the three isoforms in PL were related to those in AH or to AL.

**Conclusions:**

The levels of TGF-*β*1 and TGF-*β*3 in AH were more strongly associated with the severity of myopia than the types of TGF-*β* in PL.

## 1. Introduction

According to a recent epidemiological study, the prevalence of myopia in the global population has increased to 50% over the past 40 years, and 10% of the world population were predicted to have high myopia [1]. Hence, its prevention is an important global issue [1]. Myopia involves scleral remodeling and excessive axial length (AL) elongation [2], which can lead to posterior staphyloma, choroidal neovascularization, and tractional maculopathy [3]. This can result in pathologic myopia complications such as glaucoma, retinal detachment, chorioretinal atrophy, and macular hole, which can lead to impaired vision and even blindness [4].

The mechanism governing the development of myopia is complicated, but there are two prevailing theories: the active sclera remodeling theory [5] and the local retinal region control theory [6]. The scleral extracellular matrix (ECM) is hypothesized to be altered by the signaling cascade initiated by the blurring of the retinal image [7]. Scleral remodeling is accompanied by decreased ECM secretion and increased ECM degradation [8, 9], which ultimately leads to axial elongation. Among a number of candidate cytokines that regulate ECM remodeling [10], the multifunctional transforming growth factor-beta (TGF-*β*) plays a crucial role [11].

In mammals, TGF-*β* has three isoforms: TGF-*β*1, TGF-*β*2, and TGF-*β*3 [12, 13]. TGF-*β*s are part of a large family of polypeptides, playing important roles in cell growth and differentiation, wound healing, immune regulation [14], and the formation of ECM [15]. The TGF-*β* signaling pathway is closely associated with myopia in animal models [16]. For example, in form-deprivation myopia (FDM), the mouse TGF-*β* pathway is enriched in the eyes [17]. Seko et al. [18] observed that, in FDM chicks, TGF-*β*2 expression significantly increased in both the retina and the sclera. Jobling et al. [19] found that, in the sclera of tree shrews with induced myopia, the levels of the three isoforms of TGF-*β* decreased with axial elongation. Our previous studies have shown that the level of TGF-*β*2 in aqueous humor (AH) is positively correlated with both AL [20] and tissue inhibitors of metalloproteinase- (TIMP-) 1 and TIMP-3 [21].

Although the blood-aqueous barrier and the blood-retinal barrier make the eye a relatively isolated organ, some studies on systematic metabolism disorder have shown that changes in the levels of TGF-*β*s in plasma (PL) are related to some ocular disorders [22, 23]. For example, the level of TGF-*β*2 in PL increases with a high prevalence of congenital ectopia lentis in patients with the Marfan syndrome [22]. Guo et al. reported that a mutation in SLC39A5 that can induce the downregulation of the TGF-*β* Smad1 pathway was associated with familial high myopia [24]. Yet the relationships between the levels of TGF-*β*s in PL and myopia formation have not been completely characterized.

Therefore, in this study, we measured the levels of TGF-*β*s in PL and AH concurrently to determine whether there are relationships among their concentrations in myopic patients and between TGF-*β*s and AL.

## 2. Methods

### 2.1. Patients and Inclusion Criteria

This study was a cross-sectional study. In this study, 38 patients who underwent ICL implantation surgery from January 2018 to October 2018 were included. The inclusion criteria were as follows: aged between 18 and 45 years; normal intraocular pressure (IOP), spherical equivalent refraction (SER) between -6.00 D and -27.0 D, and AL > 24.0 mm. The exclusion criteria were systemic and metabolic diseases (e.g., diabetes, cancer, allergy, HBP, hepatitis, and hematological diseases), severe eye diseases (e.g., maculopathy and glaucoma), and ocular surgical history.

All patients received a comprehensive ocular examination, including slit lamp and dilated fundus exams. SER was examined using an open-field autorefractor (SRW500; Shin-Nippon Ophthalmic Instrument, Tokyo, Japan). AL was measured using a Zeiss IOL Master laser interferometer (Optical Biometry, IOL Master; Carl Zeiss Meditec AG, Jena, Germany).

The 38 patients were divided into three groups based on AL: group A (AL ≤ 26 mm), group B (26 mm < AL ≤ 28 mm), and group C (AL > 28 mm). All patients were sufficiently informed and signed informed consent forms, and the procedures were approved by the Ethics Committee of Shanghai Ninth People's Hospital affiliated to Shanghai Jiao Tong University School of Medicine (application number: SH9H-2018-T10-1). This study adhered to the tenets of the Declaration of Helsinki.

### 2.2. Sample Collection and Measurement of TGF-*β* Levels

Human blood and AH samples were acquired during ICL implantation surgery. The AH samples (0.1–0.2 mL) were aspirated from a central anterior chamber by paracentesis using a 26-gauge needle. The blood samples (3–4 mL) were collected 5–10 min before the surgery. All of the samples were immediately transferred to the laboratory in an iced box. Blood samples were transferred to 2.0 mL Eppendorf tubes for centrifugation at 4°C at 1000 g for 10 min to retrieve the PL samples. AH and PL samples were stored at -80°C until their measurements were processed.

A Luminex system (Luminex xMap Technology from Bio-Rad) with commercially available Milliplex xMAP kits (Millipore Corporation, Billerica, MA, USA) was used to measure the levels of each type of TGF-*β* in the samples. This technology uses multiplexed microsphere-based immunoassays, applying flow cytometric resolution to spectrally measure distinct microspheres coupled with capture molecules and reporter fluorochromes bound to detection antibodies. All assays were performed following the manufacturer's guidelines.

Human TGF-*β* Panel 2 Multiplex Assay (cat. No. TGFBMAG-64K) was used to measure each sample. The amount of TGF-*β* (pg/mL) was calculated from the standard curves for each TGF-*β* sample, according to the manufacturer's instructions.

### 2.3. Statistics

Statistical analyses were performed with SPSS 24.0 (SPSS, Chicago, Illinois, USA). All of the variables and samples were subjected to Shapiro-Wilk's tests to determine whether they were normally distributed. AL was the only normally distributed continuous variable; thus, its values are presented as the mean and standard deviation (SD), while the other continuous variables were not normally distributed and are presented in quartiles (percentile (*P*):*P*_25_, *P*_50_, *P*_75_).

As we collected samples from both eyes of one patient, we applied the generalized estimated equation (GEE) adjusted by age and sex to examine the correlations among the levels of different TGF-*β*s in AH/PL and other variables. The differences between the three groups, in terms of TGF-*β* levels, were compared using nonparametric tests (the Kruskal-Wallis test). The sex distributions of the three groups were compared using a chi-square test. Age, AL, and SER were compared among the three groups based on a one-way analysis of variance (ANOVA).

## 3. Results

### 3.1. Characteristic Information

We analyzed 59 eyes from 38 patients (11 males and 27 females) with an average age of 25.79 ± 6.97 years (range: 19–45 years) who underwent ICL implantation. The mean AL was 27.11 ± 1.27 mm (range: 24.30–30.37 mm). Overall, 10 eyes with AL ≤ 26 mm were included in group A, 37 eyes with 26 mm < AL ≤ 28 mm were included in group B, and 12 eyes with AL > 28 mm were included in group C (Table 1). The median of SER was -9.75 D. No significant differences in age or sex were detected among groups (*P* > 0.05).

### 3.2. Levels of TGF-*β*1, TGF-*β*2, and TGF-*β*3 in AH and Their Relationships with AL

**Figure 1:**
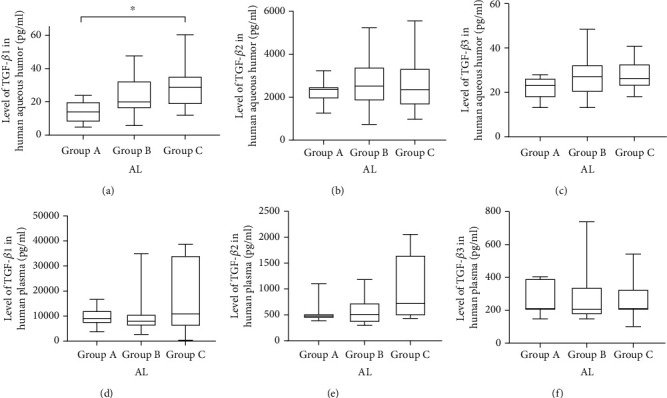
TGF-*β*1 concentrations of the three groups, detected in aqueous humor (AH) (a) and plasma (PL) (d). TGF-*β*2 concentrations in AH (b) and PL (e). TGF-*β*3 concentrations in AH (c) and PL (f). The upper and lower borders of the boxes indicate the quartiles of the TGF-*β* concentrations. The maximum and minimum values are shown by the whiskers (^∗^*P* < 0.05).

The median levels in AH were 19.97 pg/mL (range: 4.8–115.48 pg/mL) TGF-*β*1, 2446.00 pg/mL (range: 734.96–5553.00 pg/mL) TGF-*β*2, and 26.33 pg/mL (range: 13.10–57.31 pg/mL) TGF-*β*3 (Table 2). We detected significant differences among the three groups in terms of the concentrations of TGF-*β*1 in AH (Figure 1(a)), but no significant differences were detected between the concentrations of TGF-*β*2 (Figure 1(b)) and TGF-*β*3 (Figure 1(c)).

**Figure 2:**
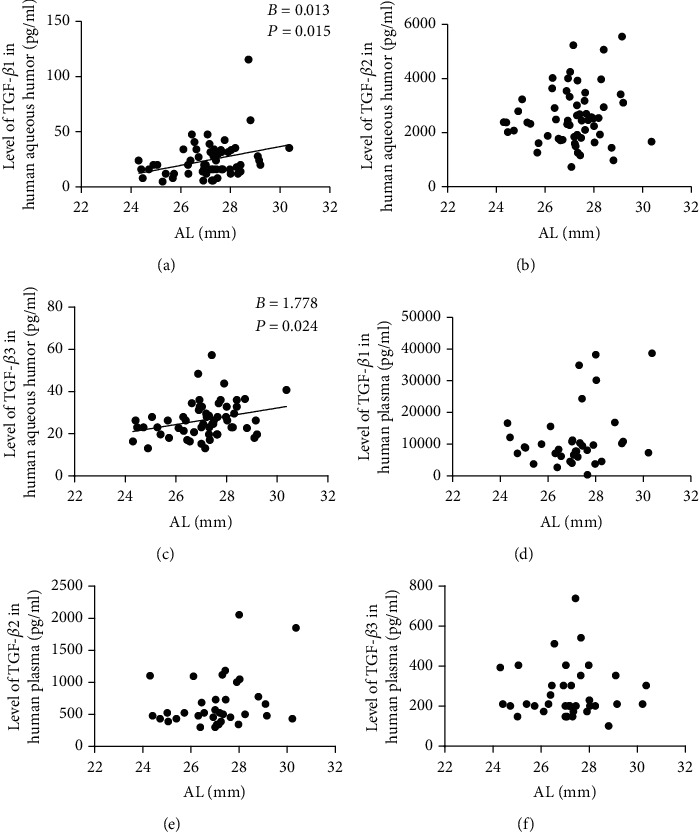
Scatter plots showing the relationships between the concentrations of TGF-*β*1, TGF-*β*2, and TGF-*β*3 in AH and AL (a, b, c) and the relationships between the concentrations of TGF-*β*1, TGF-*β*2, and TGF-*β*3 in PL and AL (d, e, f).

GEE was used to analyze the relationships between the levels of TGF-*β* in AH and AL. The *B* and *P* values are given in Table 2. The concentrations of TGF-*β*1 (Figure 2(a)) and TGF-*β*3 (Figure 2(c)) in AH were positively correlated with AL, while no significant relationship was observed between TGF-*β*2 (Figure 2(b)) in AH and AL.

### 3.3. Levels of TGF-*β* in PL and Their Relationships with AL

The concentrations of TGF-*β*s in PL are summarized in Table 3. No significant differences were detected among the three groups (Figures 1(d)–1(f)), and no relationship between the concentrations of TGF-*β*s in PL and AL was found (Figures 2(d)–2(f)).

### 3.4. Relationships between TGF-*β* Concentrations in AH and PL

**Figure 3:**
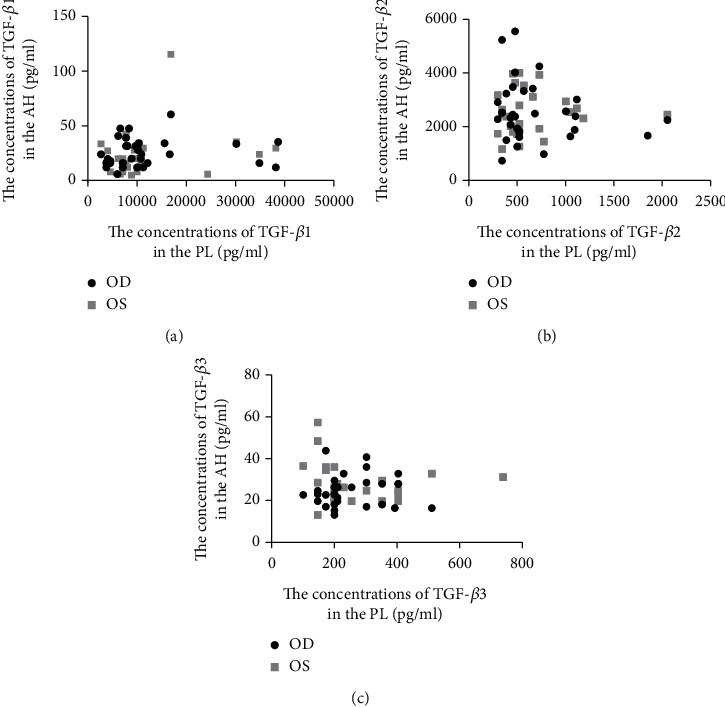
Relationships between the concentrations of TGF-*β*1 (a), TGF-*β*2 (b), and TGF-*β*3 (c) in AH and PL.

GEE indicated no significant relationships between the TGF-*β* concentrations of any type in AH and PL (Table 4, Figure 3).

## 4. Discussion

There were three major findings from this study. First, we detected a positive correlation between the levels of TGF-*β*1 (*B* = 0.013, *P* = 0.015) and TGF-*β*3 (*B* = 1.778, *P* = 0.024) in AH and AL. Second, among the three isoforms of TGF-*β*, TGF-*β*2 had the highest concentration in AH. However, they were not correlated with AL (*B* = 138.858, *P* = 0.248). Third, the levels of TGF-*β* in PL were not correlated with those in AL.

TGF-*β* is a multifunctional cytokine that regulates the growth and differentiation of cells. It regulates the proliferation of scleral fibroblast cells and production of ECM [25] and is considered the key factor mediating scleral remodeling during the development of myopia [18, 19]. Although the three isoforms are very similar in structure, they have different functions [26].

TGF-*β*1 and TGF-*β*3 bind to receptors and signal in a similar manner. They interact with transforming growth factor-*β* receptor II (T*β*RII), and subsequently, T*β*RI is recruited to the receptor complex. However, TGF-*β*2 binds very weakly to T*β*RII alone, and requires T*β*RIII to activate the complex [27, 28].

The proportions of the isoform expression of scleral TGF-*β* have been found to follow TGF − *β*1 : TGF − *β*2 : TGF − *β*3 = 2 : 33 : 1 [29]. In our study, in the AH of myopia patients, the isoform expression of scleral TGF-*β* was 0.76 : 94 : 1. In the PL of myopia patients, the isoform expression of TGF-*β* was 43 : 2 : 1. According to Jobling et al. [29], all of the three mammalian isoforms of TGF-*β* are downregulated in the sclera only 1 day after the development of myopia. Another study showed that the levels of these isoforms are not altered in the mammalian retina or choroid [30]. This implies that TGF-*β* specifically signals scleral remodeling when the signaling cascade reaches the sclera [11].

TGF-*β*1 is an important signaling molecule in the modulation of ECM during ocular development. Nevertheless, its function is controversial. Zhou et al. [31] observed that, among the three isoforms in mouse sclera, only TGF-*β*1 exhibited significant differential expression, more than threefold, during ocular development, while the expression of the others only changed marginally. In a study on tree shrews, TGF-*β*1 expression decreased by 32% 1 day after FDM formation [29]. In another study on an FDM guinea pig model, TGF-*β*1 also significantly decreased, and was found to participate in the Wnt3/*β*-catenin signaling pathway, and to mediate type I collagen-dominated ECM in the sclera [32]. In the present study, we found that levels of TGF-*β*1 in AH were positively associated with AL in adult myopic patients. This result seems to contradict previous studies [29, 32]. However, in an FDM chick model, Rohrer and Stell [33] observed that TGF-*β*1 was a potent inhibitor of basic fibroblast growth factor, which restrains the progression of myopia. However, supplementation of TGF-*β*1 did not increase myopia or induce myopia in unoccluded eyes. This implies that TGF-*β*1 may need to act with cofactors to induce myopia.

In chick models, TGF-*β*2 is a “go signal” in the development of FDM. However, there are inconsistencies in its expression during myopia formation. In guinea pigs, Li et al. [34] found that FDM decreased retinal and choroidal TGF-*β*2 mRNA and protein expression levels. Other studies have reported that TGF-*β*2 content increases or does not significantly change [18, 30, 35, 36]. In our study, we did not find a significant correlation between the levels of TGF-*β*2 and AL. Jia et al. [20] reported a positive relationship between human AH levels of TGF-*β*2 and AL. However, the subjects in that study had an average age of 67.0 ± 11.7 years, while our subjects had an average age of 25.79 ± 6.97 years. On the other hand, in Zhuang et al. [37], the vitreous level of TGF-*β*2 was not significantly different in patients with high myopia, compared to a control group. Chen et al. [25] studied different portions of sclera in guinea pigs during the induction time of lens-induced myopia (LIM), and they found that the activity of TGF-*β*2 was first elevated at the posterior pole, then in the anterior portion. This discrepancy could be due to differences in patient age. Hence, we hypothesize that the concentration of TGF-*β*2 is altered in different subjects.

Compared to TGF-*β*1 and TGF-*β*2, TGF-*β*3 has a rather low ocular concentration. There have been few studies about the implications of TGF-*β*3 in myopia formation. Jobling et al. [29] reported that TGF-*β*3 expression significantly decreases between 1 and 5 days after FDM. Although TGF-*β*3 has similar structures to TGF-*β*1 and TGF-*β*2, they have different functions. TGF-*β*3 was found to reduce the expression of alpha-smooth muscle actin (*α*-SMA) [38] in rabbit corneal tissue, a marker of fibrogenic cells. Guo et al. [26] reported that TGF-*β*3 upregulates Smad7 expression, which is an antagonist of TGF-*β* signaling and is associated with decreased fibrosis. By adding either TGF-*β*1 or TGF-*β*3, they observed differential expression of the matrix metalloproteinase 1 gene, an important regulator of ECM. In our study, we found that levels of TGF-*β*3 in AH were positively associated with AL in myopic patients. As TGF-*β*1 and TGF-*β*3 seemed to have opposite effects on fibrosis, further studies are needed to elucidate their functions in the development of myopia.

TGF-*β* is involved in two competing mechanisms of myopia formation. On the one hand, decreasing TGF-*β* levels reduce *α*-SMA expression and contraction during myopia formation [19]. On the other hand, decreases in TGF-*β* cause concurrent reductions in ECM production and scleral thickness [29]. In one study, mimicking the decreases in TGF-*β* levels during myopia induction caused a 15% reduction in collagen synthesis [29]. In another, supplementing TGF-*β*1 by intravitreal injection increased type I collagen expression [39]. Gao et al. [40] reported that the TGF-*β*1 gene was bidirectionally regulated during the induction and recovery time of LIM. It can increase or reduce tissue fibrosis via the regulation of Smad3 or Smad2/Smad7 [41], but these relationships and the manipulation of this signaling pathway need further research.

In this study, we recruited adult myopic patients with a stable refraction for at least 2 years (the changes of refraction were below 0.50 D). In this way, we studied the profile of TGF-*β* isoforms in AH and PL in a relatively stationary stage of myopia. One of the advantages of our study is that we minimized the influence of age and systemic changes on levels of TGF-*β*s. Jia et al. [20] found that the concentration of TGF-*β*2 was negatively associated with age, while Yamamoto et al. [42] found an opposite result. Nonetheless, older patients tend to take more medications for chronic conditions than younger people do. The advantage of our study is that we recruited young patients with an average age of 25.79 ± 6.97 years. Moreover, we simultaneously collected samples of AH and PL and analyzed the concentrations of TGF-*β*s. No association was found between the levels of TGF-*β*s in AH and PL. Thus, we assumed that systemic levels of TGF-*β*s may not directly influence the refractive state of healthy myopic subjects.

The limitations of our study also need to be discussed. Firstly, we recruited neither healthy young people with normal AL because of the ethical concerns of AH sample collection, nor the elder subjects (people older than 45 years) with normal AL who underwent cataract surgery because age might lead to changes of TGF-*β* levels. The patients with age-related cataract who underwent phacoemulsification were mostly of older age. They possibly have a higher prevalence of metabolic diseases (such as diabetes [43] and liver diseases [44]). Moreover, Zhu et al. [45] found that in cataract patients, the concentration of TGF-*β*2 decreased while nuclear color darkens. Besides, Yamamoto et al. [42] found that the concentration of TGF-*β*2 varied in a negative trend while aging and in different types of cataract. TGF-*β* was reported to be one of the most important cytokines inducing subcapsular cataract and posterior capsule cataract [46]. So it is note-worthy that the levels of TGF-*β*2 can be changed because of the abnormal metabolism of the lens and the capsule membrane. The relationship between the level of TGF-*β*2 in AH with age still remains undetermined: Yamamoto et al. [42] found out that the level of TGF-*β*2 decreased while the subjects were aging, while Jia et al. [20] found no correlation. Concerning the reasons above, we classified the myopic patients into three groups according to their AL and compared the changes of TGF-*β*s in these groups instead of comparing with the control group. Secondly, in this study, we did not detect the activity of the TGF-*β*s although we truly agree that their functions were related with the activity. Our further study will focus on the activity and function of TGF-*β*s in myopic eyes. Thirdly, the refraction state of the subjects in this study was in a relatively stationary stage, which means we could not discern the level changes of TGF-*β* isoforms during myopia formation.

## 5. Conclusion

In conclusion, we found that the concentrations of TGF-*β*1 and TGF-*β*3 in AH were positively associated with AL in myopic young patients, while the concentrations of TGF-*β*s in PL were not statistically correlated with AL. This indicates that TGF-*β*1 and TGF-*β*3 could be potential targets for myopia control. Further study is required to elucidate the molecular mechanisms related to TGF-*β*s in the progression of myopia.

## Figures and Tables

**Table 1:** Sample characteristics.

	Group A	Group B	Group C	Total	*P*
N (patients, eyes)	7, 10	22, 37	9, 12	38, 59	
Age (years, mean ± SD)	25.43 ± 6.079	26.68 ± 8.312	23.89 ± 3.01	25.79 ± 6.968	0.604
Sex (male : female)	1 : 6	7 : 15	3 : 6	11 : 27	0.783
SER (*D*, *P*_50_, *P*_25_, *P*_75_)	-7.44, -8.28, -6.34	-9.50, -11.25, -8.63	-13.13, -14.69, -10.88	-9.75, -12.00, -8.50	<0.001
AL (mm, mean ± SD)	24.99 ± 0.521	27.17 ± 0.510	28.72 ± 0.683	27.11 ± 1.265	<0.001

SER: spherical equivalent refraction; AL: axial length. One-way ANOVA test. Pearson's chi-square test.

**Table 2:** Concentrations of TGF-*β*s (pg/mL) in AH and relationship with AL (*P*_50_ (*P*_25_, *P*_75_)).

	Group A (*n* = 10)	Group B (*n* = 37)	Group C (*n* = 12)	Total (*n* = 59)	*B* value	*P* value
TGF-*β*1	13.94 (7.90, 19.97)	19.97 (14.95, 32.50)	29.67 (19.97, 35.27)	19.97 (13.94, 31.56)	0.013	0.015
TGF-*β*2	2352.00 (1920.75, 2489.25)	2462.00 (1827.50, 3400.50)	2556.50 (1646.50, 3341.25)	2446.00 (1809.00, 3178.00)	138.858	0.248
TGF-*β*3	23.03 (17.64, 26.33)	26.33 (20.25, 32.02)	26.33 (22.78, 36.40)	26.33 (19.72, 31.21)	1.778	0.024

GEE: generalized estimating equation; *B* value: coefficient variables.

**Table 3:** Concentrations of TGF-*β*s (pg/mL) in PL and relationship with AL (*P*_50_ (*P*_25_, *P*_75_)).

	Group A *n* = 7	Group B *n* = 22	Group C *n* = 9	Total *n* = 38	Spearman's test^†^		*P* value
					*R*	*P*	
TGF-*β*1	9104.00 (7101.00, 12163.00)	7753.00 (5270.50, 11033.50)	10551.00 (7899.50, 26861.25)	8984.00 (6295.75,11939.50)	0.193^†^	0.259	0.431
TGF-*β*2	477.84 (432.53, 523.44)	523.44 (365.44, 912.61)	718.69 (483.53, 1038.50)	523.44 (432.53, 947.05)	0.236^†^	0.172	0.507
TGF-*β*3	210.47 (200.75, 393.08)	229.90 (186.99, 378.40)	205.61 (180.11, 279.91)	210.47 (200.75, 340.27)	0.203^†^	0.907	0.277

Spearman's rank coefficient^†^. Independent-samples Kruskal-Wallis's test.

**Table 4:** Relationships between TGF-*β* concentrations (pg/mL) in AH and PL (*P*_50_ (*P*_25_, *P*_75_)).

	AH-OD	AH-OS	PL	GEE	
				*B* value	*P* value
TGF-*β*1	19.97 (15.96, 33.75)	19.97 (10.92, 29.68)	6295.75 (8984.00, 11939.50)	0.023	0.073
TGF-*β*2	2388.0 (1810.5, 3279.0)	2454.5 (1791.5, 3125.5)	432.53 (523.44, 947.05)	-0.000009	0.398
TGF-*β*3	24.62 (18.89, 29.07)	26.33 (22.47, 33.26)	200.75 (210.47, 340.27)	0.008	0.881

OD: right eyes. OS: left eyes. PL: plasma. GEE: generalized estimated equation. *B* value: coefficient variables.

## Data Availability

The data have not been placed in any online data storage. The datasets generated and analyzed during the study are available upon request from the first author.
